# Anode interfacial layer formation via reductive ethyl detaching of organic iodide in lithium–oxygen batteries

**DOI:** 10.1038/s41467-019-11544-8

**Published:** 2019-08-07

**Authors:** Xiao-Ping Zhang, Yi-Yang Sun, Zhuang Sun, Chu-Shu Yang, Tao Zhang

**Affiliations:** 1 State Key Lab of High Performance Ceramics and Superfine microstructure, Shanghai Institute of Ceramics, Chinese Academy of Sciences, 1295 Dingxi Road, 200050 Shanghai, People’s Republic of China; 2 Center of Materials Science and Optoelectronics Engineering, University of Chinese Academy of Sciences, 100049 Beijing, People’s Republic of China

**Keywords:** Energy storage, Electrochemistry, Energy science and technology

## Abstract

As soluble catalysts, redox mediators can reduce the high charging overpotential of lithium-oxygen batteries by providing sufficient liquid-solid interface for lithium peroxide decomposition. However, the redox mediators usually introduce undesirable reactions. In particular, the so-called “shuttle effect” leads to the loss of both the redox mediators and electrical energy efficiency. In this study, an organic compound, triethylsulfonium iodide, is found to act bifunctionally as both a redox mediator and a solid electrolyte interphase-forming agent for lithium-oxygen batteries. During charging, the organic iodide exhibits comparable lithium peroxide-oxidizing capability with inorganic iodides. Meanwhile, it in situ generates an interfacial layer on lithium anode via reductive ethyl detaching and the subsequent oxidation. This layer prevents the lithium anode from reacting with the redox mediators and allows efficient lithium-ion transfer leading to dendrite-free lithium anode. Significantly improved cycling performance has been achieved by the bifunctional organic iodide redox mediator.

## Abstract (web summary)

To fulfill the great promise of Li-O_2_ batteries, the high charge overpotential is a major challenge that has to be addressed. Here the authors introduce triethylsulfonium iodide as a redox mediator as well as an enabler of a protective layer on Li anode, leading to notable electrochemical performance.

## Introduction

Lithium metal with high theoretical specific capacity (3860 mAh g^−1^) and the lowest negative electrochemical potential (−3.04 V vs. SHE) has been extensively studied for next-generation lithium metal batteries including Li–S, Li–O_2_, and solid-state Li-metal batteries. A lithium metal anode faces two major challenges: the formation of a stable solid electrolyte interphase (SEI) and the ability to electrodeposit lithium non-dendritically. From the electrochemical point of view, the lithium metal SEI layer must permit fast Li-ion transport while simultaneously block electron transfer through the SEI layer. This is of paramount importance for establishing a completely reversible Li^+^/Li charge transfer process. The SEI components determine the rechargeable ability of lithium metal anode, as reflected by Coulombic efficiency, cycling performance, as well as dendrite growth. Intensive research has focused on modifying lithium metal anode by an artificial scaffold to enhance the stability of the spontaneously formed SEI layer^1–6^. Such approaches, called interfacial engineering, are typically realized by modifying the salts in electrolytes^1,4^, inorganic Al_2_O_3_ or AlF_3_ framework^2,5^, double polymer network^3^, and hard solid-state ceramics^6^. These attempts have successfully advanced the lithium metal anode technique. Meanwhile, significant interest has been spurred in studying the formation and degradation mechanism of SEI components related to the complexity of electrolytes with different solvents, solutes, and shuttled active species. For instance, (1) how to rationally control the organic components in a common organic-inorganic hybrid SEI layer; (2) how the SEI layer evolves under the interaction with shuttled oxygen intermediates, polysulphides or redox mediators (RMs). These questions still remain to be resolved.

Oxygen species, including O_2_ radical and LiO_2_ intermediates are inevitable when we consider the SEI-related issues in Li–O_2_ batteries^7–9^. At the same time, reducing large overpotential upon charging is a key issue in the further development of Li–O_2_ batteries^10,11^. Recently, dissolved RMs were proposed as molecular shuttles to promote the charge transfer between Li_2_O_2_ surface and air cathode that resulted in accurate charging at moderate voltages^12^. Up to now, researchers have studied several RMs in Li–O_2_ cells. Chen et al. combined a solution of tetrathiafulvalene (TTF/TTF^+^) in DMSO with a nanoporous gold cathode, which could reduce the charging overpotential^12^. Lim et al. introduced lithium iodide (I_3_^−^/I_2_) redox couple in tetra(ethylene)glycol dimethyl (TEGDME), which exhibited stable cycle performance^13^. Other RMs, such as iron phthalocyanine (FePc)^14^, 2,2,6,6-tetramethylpiperidinyloxy(TEMPO)^10^, tris[4-(diethylaminophenyl]-amine(TDPA)^15^, cobalt bis(terpyridine)-(Co(Terp)_2_)^16^, and N-methyl-phenothiazine (MPT)^17^ were also suggested. The incorporation of RMs efficiently reduces charge polarization. However, side reactions accompanied by the RMs lead to the degradation of the catalytic activity, which results in gradually increased charging overpotential after a certain number of cycles^18^.

The increased charging overpotential has been considered to be a shuttle effect related to the diffusion of soluble RMs during the charging process^19^. Oxidized RMs could diffuse through the separator and be reduced at the Li metal anode, leading to the deactivation of the RMs and the decline of electrical energy efficiency during charging process^20^. To avoid the shuttle effect, solid-state electrolytes and modified separators were suggested to protect the Li anode from the side reactions to achieve high cycling performance^18,21–23^. However, these ex situ protective layers suffer from interface separation after repeated cycling. In our previous study, we proposed an in-situ method by introducing indium triiodide (InI_3_) to suppress the shuttle effect. Pre-depositing indium layer on the Li surface efficiently prevents the RMs from being reduced at the Li anode^24^. Meanwhile, we noticed that this inorganic metallic protective layer is an electronic conductor. An indium-lithium alloying process and minor lithium loss still happen at every charging process.

Here we propose organic iodides as bifunctional RMs for Li–O_2_ batteries. These organic iodides not only reduce the charging overpotential, but also represent a novel strategy to protect the Li anode from the I_3_^–^ attack. During charging, the organic cation in the TEGDME electrolyte deposits on the surface of the Li anode, in situ forming a thin organic film, serving as a SEI-like layer. Different from the previous metallic indium layer, this organic layer is not only a Li-ion conductor, but also an electron insulator. Meanwhile, the SEI-like layer contains organic compounds, which exhibits good flexibility and strong affinity to the Li metal surface. In this case, it is possible that the organic iodide prevents the lithium anode from being attacked by the soluble I_3_^–^, while retaining its RM function. There is a great variety of organic iodide compounds, such as triethylsulfonium iodide (Supplementary Fig. 1), choline iodide, tetramethylammonium iodide, trimethylsulfoxonium iodide, et al. In the Supporting Information (SI), we provide a partial list of organic iodides in Supplementary Fig. 2.

In this work, we find that triethylsulfonium iodide (C_6_H_15_SI, TESI) exhibits bifunctional behavior, i.e., acting as both an efficient RM and in situ SEI-like layer forming agent on lithium anode through reductive ethyl detaching and subsequent oxidation. The SEI-like layer contains organic and inorganic components, which efficiently prevent the attack of soluble I_3_^–^ and transport Li-ions. When the TESI additive is added, the battery yields stable cycling performance and high energy efficiency.

## Results

### In-situ formation of SEI-like layer on Li anode

**Fig. 1:**
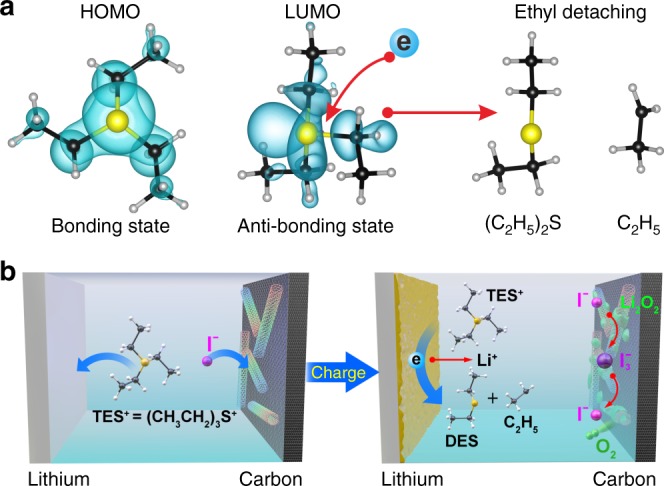
In-situ formation process of SEI-like layer on Li anode. **a** Calculated HOMO and LUMO orbitals of a TES^+^ cation. **b** Schematic illustration of the in-situ formation process of SEI-like layer on Li anode during charging

Choosing TESI in this work was based on the following considerations: (1) InI_3_ has been demonstrated to be an effective bifunctional RM except the unwanted alloying of In and Li metal; (2) In the oxidation environment, alkyl group is easily oxidized into lithium carboxylate, which is a well-known component in SEI^25^. A thin film on Li anode based on lithium carboxylate serves as an insulating SEI-like layer. Our density functional theory calculation suggests that the triethylsulfonium cation (TES^+^) could provide the required ethyl group in a reductive environment. Figure 1a shows the calculated HOMO and LUMO orbitals of a TES^+^ cation. While HOMO is a symmetric bonding orbital, LUMO exhibits a clear nodal surface between one ethyl group and the remaining part of the cation. When acquiring an electron from the Li anode, the occupation of the LUMO will promote the detaching of that ethyl group from TES^+^ forming diethyl sulfide C_4_H_10_S (DES) and an ethyl radical. The reductive ethyl detaching reaction is:

1 $${\mathrm{Li}}\left( {{\mathrm{metal}}} \right) + {\mathrm{TES}}^ + \to {\mathrm{DES}}\left( {{\mathrm{neutral}}} \right) + {\mathrm{Li}}^ + + {\mathrm{CH}}_3{\mathrm{CH}}_2$$

The detaching ethyl radical is an unstable component, which could be easily oxidized by O_2_ and O_2_^−^. We will discuss the reactions of the ethyl radical in the later sections. Figure 1b shows a schematic illustration of the in-situ formation process of the organic film. According to this scheme, I^−^ anions are released by the organic iodide. During repeated cycling, the I^−^ anions are oxidized to I_3_^−^ anions, which act as RM in electrolyte and decrease the charging overpotential. At the same time, the organic cations (TES^+^) diffuse towards the Li metal, where chemically reductive reaction occurs spontaneously between the TES^+^ and the Li metal. These reactions in situ form a SEI-like layer associated with DES and CH_3_CH_2_· radical on the surface of the Li anode, which protects the Li anode in Li–O_2_ battery. The organic film conducts Li-ion efficiently and meanwhile blocks electrons.

### Electrochemical performance of TESI-containing Li–O_2_ cell

**Fig. 2:**
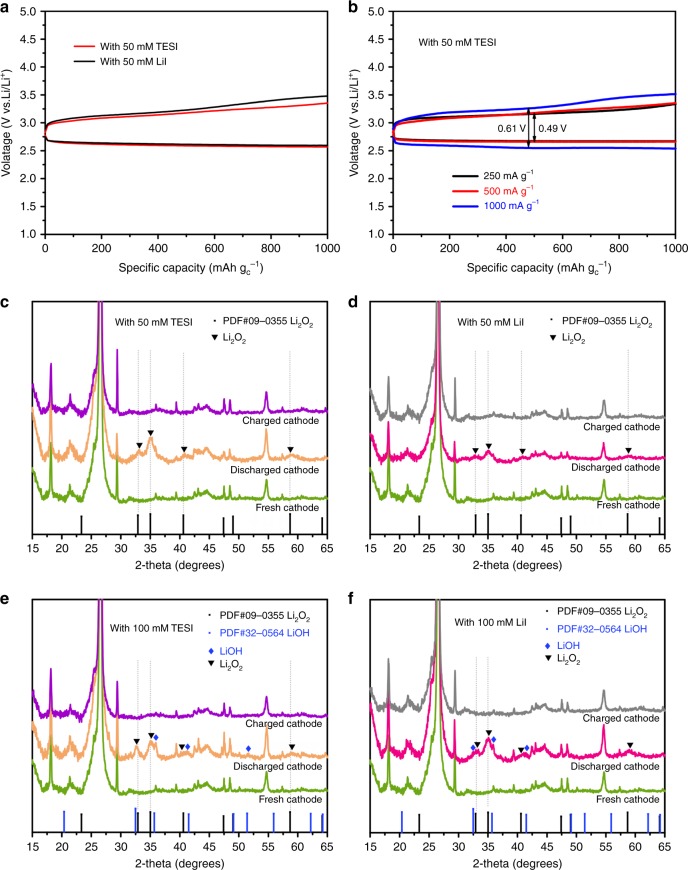
Electrochemical performance and XRD patterns of the Li-O_2_ cells with TESI or LiI additives. **a** Galvanostatic discharge/charge profile at 500 mA g^−1^ with 50 mM TESI or 50 mM LiI additives at a limited specific capacity of 1000 mAh g^−1^. **b** With 50 mM TESI additive for the first cycle, current density: 250, 500 and 1000 mA g^−1^. XRD patterns of the cathode at the end of discharge and the cathode at the end of charge with (**c**) 50 mM TESI additive, (**d**) with 50 mM LiI additive, (**e**) with 100 mM TESI additive, (**f**) with 100 mM LiI additive

The Li–O_2_ cell used in this work was composed of a Li anode, a porous cathode consisting of single walled carbon nanotubes-single layer graphene (SWNTs-SLG), a 1.0 M LiTFSI/TEGDME electrolyte with 50 mM LiI or TESI additives. Discharge and charge profiles for the Li-O_2_ cell with 50 mM TESI at a current density of 500 mA g^−1^ are presented in Fig. 2a. Data for the Li–O_2_ cell with 50 mM LiI are provided for comparison. The TESI-containing cell shows a discharge plateau at about 2.7 V, which is similar to that of the LiI-containing cell. Moreover, the charging overpotential of the TESI-containing cell is even slightly lower than that of the LiI-containing cell. These results indicate that I_3_^−^ and I_2_ in the TESI-containing Li–O_2_ cell act as a redox couple efficiently, similar to LiI. Figure 2b shows the discharge/charge curves of Li–O_2_ batteries with 50 mM TESI additive at a current density of 250, 500, and 1000 mA g^−1^ for the first cycle. The charging voltage platform at 1000 mA g^−1^ (0.61 V) is just slightly higher than that at 250 and 500 mA g^−1^ (0.49 V). A large change in current density with a small shift in the voltage platform demonstrates the outstanding oxygen evolution reaction performance and electrochemical stability of the TESI.

Figures 2c–f show the XRD patterns from the cathode discharged to 1.8 mAh capacity and then charged with 50 mM TESI or LiI. For comparison, the same experiments were carried out with 100 mM TES and LiI. With 50 mM TESI or LiI, the cells mainly exhibited the formation and decomposition of Li_2_O_2_. When concentrated TESI or LiI (100 mM) were used, weak LiOH diffraction peaks appeared. The discharge products in the TESI-containing Li–O_2_ cell are consistent with those in the LiI-containing cell.

**Fig. 3:**
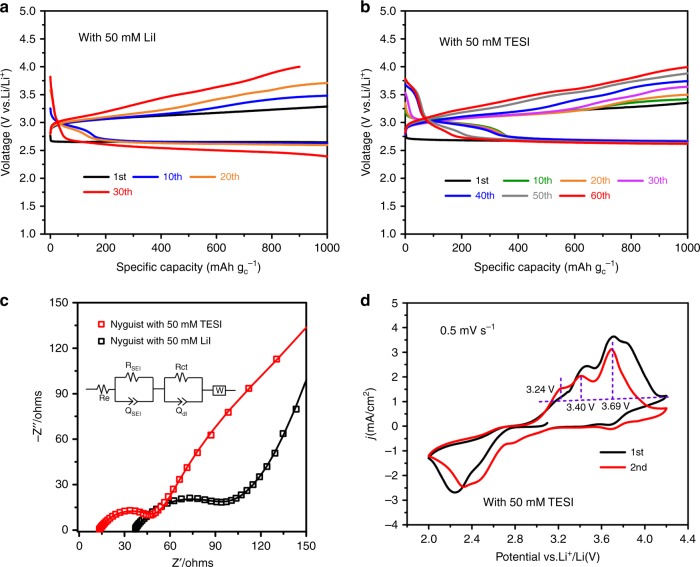
Electrochemical performance of the Li-O_2_ cells with TESI or LiI at a limited specific capacity of 1000 mAh g^−1^. **a** With 50 mM LiI additive for 30 cycles, current density: 500 mA g^−1^. **b** With 50 mM TESI additive for 60 cycles, current density: 500 mA g^−1^. **c** EIS of the Li-O_2_ cell after 30 cycles with 50 mM TESI or 50 mM LiI and EIS analysis using an equivalent circuit model (shown in the inset). **d** Cyclic voltammograms (CV) of the TESI-containing Li-O_2_ Cell

The cycling performance of the TESI-containing and LiI-containing Li–O_2_ cells is shown in Fig. 3a, b. The discharge capacities of the electrodes are limited to 1000 mAh g^−1^. In Fig. 3b, the theoretical specific capacity delivered by the I^–^/I_3_^–^ redox couple is 363.63 mAh g^−1^. The measured specific capacity of the I^–^/I_3_^–^ redox couple is 340.1 mAh g^−1^ in the 50 mM TESI-containing Li–O_2_ cell. The efficiency of releasing I^–^ by TESI is 93.52%. Compared with the LiI-containing Li–O_2_ cell (Fig. 3a), there are two beneficial results achieved from the TESI-containing cell (Fig. 3b). One is the improved cycling performance, which is superior to that of the LiI-containing one. The cell with 50 mM TESI exhibits excellent cycle stability over 60 cycles of discharging/charging with relatively small overpotential. The other benefit is the increased discharge potential at the later part of discharging. The discharge median voltage of the first cycle is above 2.7 V. Even after 60 cycles the voltage is still higher than 2.61 V. In contrast, the cell with LiI can only work stably for 30 cycles, and the discharge median voltage was at the 30th cycle just 2.42 V (Fig. 3a). These provide an electrochemical evidence for our design, suggesting that the in-situ formation of organic compound-containing film effectively protects the Li metal anode from the attack of the soluble I_3_^−^, reducing the extra consumption of the I_3_^–^ ions during charging and improving electrochemical performance.

The analysis of electrochemical impedance spectra (EIS) (Fig. 3c and Supplementary Fig. 2) indicates that the TESI-containing Li–O_2_ cell exhibits considerably smaller interfacial resistance than the LiI-containing cell. In order to avoid the influence of the cathode products, the Li−O_2_ cells after 30 cycles with 50 mM TESI or 50 mM LiI were both reassembled with pristine air cathodes. Supplementary Table 1 shows the EIS of the Li–O_2_ cells reassembled after 30 cycles. The TESI-containing Li–O_2_ cell consistently exhibits smaller interfacial resistance than the LiI-containing cell. These results demonstrate that TESI-containing cell can achieve an efficient Li-ion transfer.

The cyclic voltammograms (CVs) of the TESI-containing Li–O_2_ cell (Fig. 3d) show reversible features at 3.24, 3.40, and 3.69 V, which correspond to the reactions LiO_2 → _O_2_ + e^− ^+ Li^+^, Li_2_O_2 _→ O_2_ + 2e^− ^+ 2Li^+^, and 3I^– ^→ I_3_^− ^+ 2e^−^, respectively. These assignments are consistent with the Li_2_O_2_ and LiO_2_ oxidation before 3.5 V and the following I_3_^−^/I^−^ redox process between 3.5 and 4.0 V, suggesting that the organic iodide possesses similar Li_2_O_2_ oxidation behavior to the inorganic LiI. Note that both the first discharge curves show a flat plateau at around 2.7 V, whereas the subsequent discharge profiles show shoulders at higher potentials. This is due to the reductive reaction of I_3_^−^ + 2e^− ^→ 3I^−^ at air cathode because it is common that part of I_3_^−^ remains after the first charging process^24^. The I_3_^−^/I^−^ discharge potential is consistent with that in Li–I_2_ cells^26^. The typical redox behavior of iodine has been well characterized by previous studies of halogen electrochemistry^26,27^. Therefore, this work will focus on revealing the functions of TES^+^ cation.

### Synergic analysis of the functions of TES^+^ cation

To gain further insight into the functions of TES^+^ cation on air cathode and Li anode, the air cathode and Li anode after cycling were investigated by scanning electron microscope (SEM), Fourier transform infrared (FTIR), gas chromatography-mass spectrometry analysis (GC-MS) and X-ray photoelectron spectroscopy (XPS). The SEM images of the fresh air cathodes are presented in Supplementary Fig. 4. The SEM images of the air cathodes after cycling in Li–O_2_ cells with TESI or LiI additives are shown in Supplementary Fig. 5. XPS spectra on the surface of the same air cathodes are shown in Supplementary Fig. 6. From the analyses of the air cathodes (Supplementary Notes 1 and 2), there is no significant function of TES^+^ cation on air cathode. Therefore, this work will focus on revealing the function of TES^+^ cation on Li anode.

**Fig. 4:**
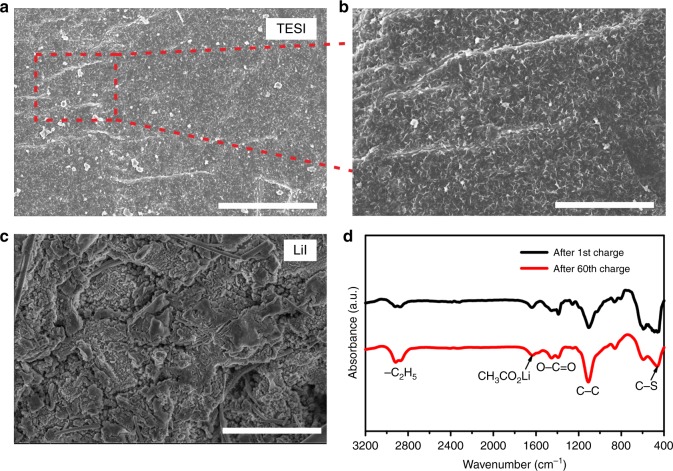
Surface analysis of the Li anodes after cycling in Li-O_2_ cells. **a**, **b** SEM images of the Li anode after the 60 cycles with 50 mM TESI. Scale bars are 4 μm (**a**) and 2 μm (**b**). **c** SEM image of the Li anode after 30 cycles with 50 mM LiI. Scale bar is 4 μm. **d** FTIR spectra on the surface of the Li anode after 1 cycle and 60 cycles with 50 mM TESI additive

The SEM image of the Li anode in the TESI-containing Li–O_2_ cell after 60 cycles shows a thin fluffy layer at the Li anode surface (Fig. 4a). This can be observed more clearly at a higher magnification (Fig. 4b). Figure 4c shows the SEM image of the Li anode surface in the LiI-containing Li–O_2_ cell after 30 cycles. In contrast, gully domains due to the lithium dissolution and ridge domains owing to the lithium deposition were clearly observed. The difference of the surface morphology suggests that the surface layer formed in the TESI-containing Li–O_2_ cell facilitates the Li-ion transfer, hence suppressing the accumulation of the Li on the surface. In addition, the SEM images of Li metal anodes in the TESI-containing and LiI-containing Li–O_2_ cells after the first cycle are provided in the SI (Supplementary Fig. 7). The observed results are consistent with that in Figs. 4 a–c.

FTIR measurements were then performed. The results are shown in Fig. 4d. The peaks at 1457 and 1390 cm^−1^ correspond to the O–C=O vibrations of Li_2_CO_3_, and the peak at 1620 cm^−1^ is attributed to CH_3_CO_2_Li^28^. The characteristic peaks at 2920 cm^−1^ and 2866 cm^−1^ correspond to the –C_2_H_5_ group^29^, and the peak at 1105 cm^−1^ arises from the C–C stretching^30^. We also observe a peak at 455 cm^−1^, which corresponds to the C–S bond^31^. The presence of the C_2_H_5_ ethyl group and C–S bonds provides direct evidence that the surface layer is mainly derived from the TES^+^ and DES, because the C_2_H_5_ and C-S bonds are their typical features.

**Fig. 5:**
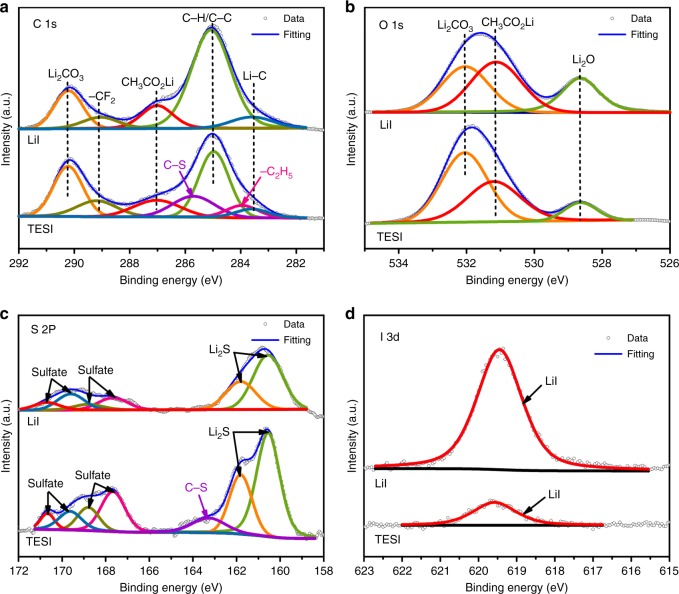
XPS spectra on the surface of Li anodes. Two samples from the Li–O_2_ cells with 50 mM TESI after 60 cycles and 50 mM LiI additives after 30 cycles were measured. **a** C 1s spectra. **b** O 1s spectra. **c** S 2p spectra. **d** I 3d spectra

XPS measurements were carried out to further confirm the element components of the surface layer in the TESI-containing Li–O_2_ batteries, as shown in Fig. 5. The XPS spectra of Li metal anodes in the TESI-containing Li–O_2_ cell after the first cycle are provided in the SI (Supplementary Fig. 8). Figure 5a shows the XPS profiles of C 1 s acquired from the Li anodes in the Li–O_2_ cell with 50 mM TESI or 50 mM LiI additives after repeated cycling, respectively. The peaks at 283.5 eV and 284.9 eV are assigned to Li–C bond and C–H/C–C group of TEGDME electrolyte^32^, and the three peaks at 287.2, 289.0, and 290.2 eV can be ascribed to CH_3_CO_2_Li, –CF_2_ and Li_2_CO_3_, respectively^33,34^. Importantly, C–S and C_2_H_5_ bonds located at 285.7 and 283.9 eV are only observed for the Li metal in the TESI-containing Li–O_2_ cell, further indicating that the surface layer is mainly derived from the TES^+^ and DES^35^. In the spectra of O 1 s (Fig. 5b), the peaks at 528.6, 531.3, and 532.0 eV are attributed to Li_2_O, CH_3_CO_2_Li, and Li_2_CO_3_, respectively^36^. In the S 2p spectrum of the Li metals (Fig. 5c), two close peaks located at 161.8 and 160.6 eV correspond, respectively, to S 2p_1/2_ and S 2p_3/2_ of chemical bonding for Li_2_S^37^, and the peak at 163.4 eV corresponds to C–S bond^38^. These results are consistent with the C 1s XPS results. The peaks at 167.5, 168.7, 169.6, and 170.8 eV can be attributed to sulfate^38^. It is worth noting that in the spectra of I 3d (Fig. 5d), the signal of LiI species in the Li anodes from the TESI-containing Li–O_2_ cell is much weaker than that in the LiI-containing Li–O_2_ cell, indicating that the TESI-derived surface layer can efficiently protect Li anode from electronic reduction with soluble triiodide.

**Fig. 6:**
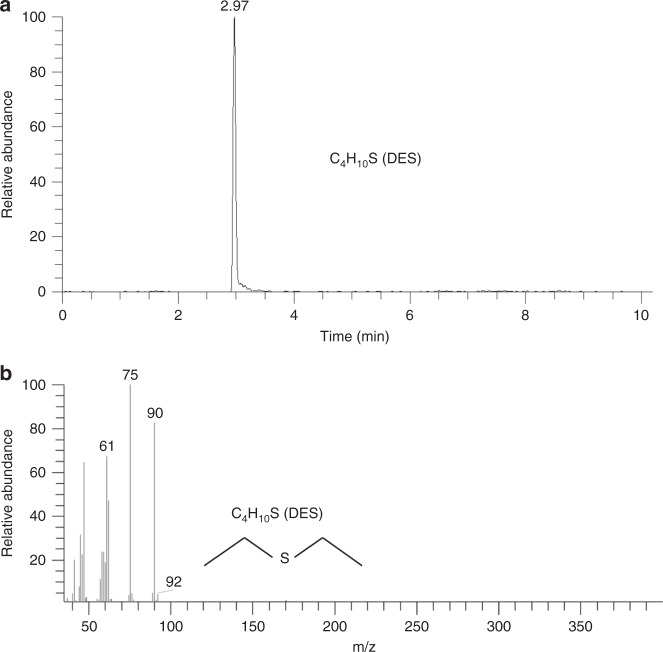
GC-MS measurement. The extracted chromatogram (*m*/*z* 90) and DES mass spectra (DES = *m*/*z* 90) were taken from the Li anode of the Li–O_2_ cell with 50 mM TESI after 60 cycles. **a** Extracted chromatogram. **b** DES mass spectra (DES = *m*/*z* 90)

The GC-MS was employed to analyze the chemical species on the surface of the Li anode with 50 mM TESI additive. In the extracted chromatogram (*m*/*z* 90) (Fig. 6a), the peak at 2.97 min is assigned to DES. The corresponding mass spectrum of the fragment peaks of DES is shown in Fig. 6b (*m*/*z* 90). It is worth noting that *m*/*z* 92 is the isotope of DES. This result provides direct evidence that DES does exist on the surface of the Li anode after 60 cycles with the TESI additive.

After characterizing the components of the SEI layer on the Li anode with 50 mM TESI, we further analyze the forming process of the SEI layer by Raman spectroscopy. The results are shown in Supplementary Fig. 9, where the peak at 600 cm^−1^ is the characteristic peak of TESI. The Raman spectra show that the characteristic peak of TESI after the first cycle is weaker than that in pristine electrolyte with 50 mM TESI. After five cycles, the characteristic signal of TESI completely disappears, indicating that the SEI layer is formed in the first five cycles and then stabilized.

The analyses above demonstrate that the SEI layer mainly consists of organic components, DES and CH_3_CO_2_Li, and inorganic components, Li_2_CO_3_, Li_2_O, and Li_2_S. The organic component DES only appears on the Li anode from the TESI-containing Li–O_2_ cell, which is the key component in the SEI layer. Organic components combined with inorganic lithium salts results in a SEI-like layer which exhibits better uniformity, ionic conductivity and flexibility than inorganic SEI layers.

## Discussion

**Fig. 7:**
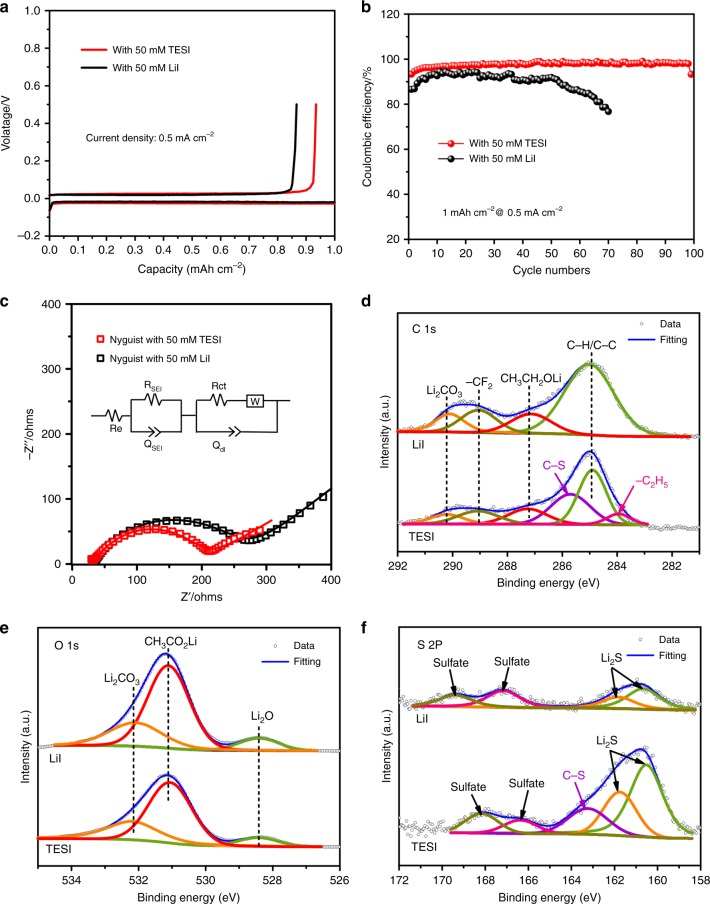
Electrochemical performance and analysis of the Li anodes from Li | Cu cells in the presence of O_2_ with a limited specific capacity of 1 mAh cm^−2^. **a** Galvanostatic discharge/charge profile at 0.5 mA cm^−2^. **b** Subsequent cycles, current density: 0.5 mA cm^−2^. **c** EIS of the Li | Cu cells after 100 cycles with 50 mM TESI or 50 mM LiI, and EIS analysis using an equivalent circuit model (shown in the inset). **d**–**f** XPS spectra of the Li anodes after 100 cycles with 50 mM TESI and 50 mM LiI additive

To evaluate the long-term stability of Li plating/stripping behavior in a battery, Li | Cu cells in the presence of O_2_ with LiI and TESI additives were assembled. For Li | Cu cells, high Coulombic efficiency (CE) is a good indicator of the formation of a SEI-like layer. In the presence of O_2_, the initial CE of the cells with LiI or TESI additives is 86.63% and 93.36% at 0.5 mA cm^−2^, respectively. After 70 cycles, the CE of LiI is evidently lower than 80%, while a remarkably high CE of 98% is achieved in the TESI-containing Li | Cu cell after 100 cycles, as shown in Fig. 7a, b. The corresponding EIS shown in Fig. 7c indicates that the TESI-containing Li | Cu cell facilities the Li-ion transfer.

In the TESI-containing Li | Cu cells, morphologies of Li anode were recorded (Supplementary Fig. 10) after 100 cycles. A thin, uniform and flexible layer without any porous structure covers the deposited lithium and effectively transports Li-ions, such that Li metal deposition takes place underneath the SEI layer without forming dendrites. XPS analysis was used to further investigate the surface components of the anode in the TESI-containing Li | Cu cell (Fig. 7d–f). In the presence of O_2_, the SEI-like layer is mainly composed of organic components (Supplementary Fig. 11 and Supplementary Note 3) involving DES, CH_3_CO_2_Li and inorganic components (e.g., Li_2_CO_3_, Li_2_O, and Li_2_S), which achieves high ionic conductivity and dendrite-free Li deposition.

**Fig. 8:**
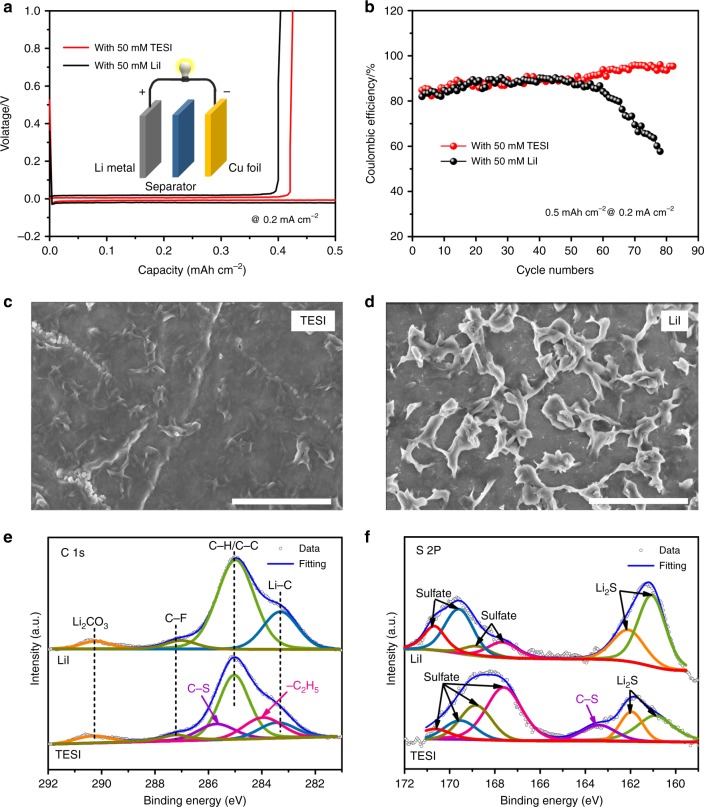
Electrochemical performance and analysis of the Li anodes of Li | Cu cells with a limited specific capacity of 0.5 mAh cm^−2^. **a** Galvanostatic discharge/charge profile at 0.2 mA cm^−2^. **b** Subsequent cycles, current density: 0.2 mA cm^−2^. **c** SEM image of the Li anode after 80 cycles with 50 mM TESI additive. Scale bar is 2 μm. **d** SEM image of the Li anode after the 80 cycles with 50 mM LiI additive. Scale bar is 2 μm. **e** C 1s XPS spectra of the Li anodes after 80 cycles. **f** S 2p XPS spectra of the Li anodes after 80 cycles

The protective behavior of the SEI-like layer has been revealed in the presence of O_2_. We further assembled gastight Li | Cu cells to study whether similar behavior exists in the absence of O_2_. In this case, the TESI-containing Li | Cu cell shows 84.8% CE in the first cycle and the CE increases gradually to over 95% after 80 cycles (Fig. 8a). However, for the LiI-containing cell, the CE value is 81.9% in the first cycle, starts to decrease after 50 cycles, and significantly decreases to only 57.7% after 80 cycles (Fig. 8b). The reasons for the fading of CE after 50 cycles are as follows: (1) the sharply increased impedance, (2) an uneven lithium surface, and (3) optically visible dead lithium on the Cu substrate (Supplementary Figs. 12 and 13). The corresponding EIS suggests that the TESI-containing Li | Cu cell facilities the Li-ion transport (Supplementary Fig. 14 and Supplementary Table 2). The surface morphology of the Li anodes after 80 cycles was also recorded. From Fig. 8c, d, it is observed that uneven growth of the electrodeposited lithium occurs in the LiI-containing Li-Cu cell, which produces Li dendrites that are exposed on the surface of Li anode. In sharp contrast, there is no clear Li dendrite formation observed on the surface of Li anode in the TESI-containing Li | Cu cell, which suggests that the deposition of lithium occurs mainly under a surface layer on the Li anode. Large-area SEM images of the Li anodes from TESI-containing and LiI-containing Li | Cu cells are shown in Supplementary Fig. 15, where a flatter surface can be observed on the TESI-containing cell than that on the LiI-containing cell.

The same surfaces as in the SEM experiment were analyzed by XPS. Fig. 8e, f show the C 1s and S 2p XPS spectra. The Li anode from the TESI-containing cell generates the XPS peak that can be assigned to C-S bond, while this peak is absent in the Li anode from the LiI-containing cell. The component of CH_3_CO_2_Li is absent in the TESI-containing cell (Supplementary Fig. 16 and Supplementary Note 4). These results suggest that the Li anode indeed reacts with the TES^+^, however, the oxidation process of the detached CH_3_CH_2_· radical cannot proceed in the absence of O_2_.

Combining all the data above, the SEI-like layer formation in the Li−O_2_ cell containing the organic iodide TESI is proposed as follows: (1) a reductive ethyl detaching process occurs at Li anode, releasing CH_3_CH_2_· radical and neutral DES molecule. The reductive process includes the chemical reaction between Li atoms and TES^+^; (2) The CH_3_CH_2_· radical react rapidly with O_2_ and O_2_^−^ to produce CH_3_CO_2_Li, and the DES molecules absorb on the surface of Li anode. The resulting surface layer not only consists of the inorganic components, such as Li_2_CO_3_, Li_2_O and Li_2_S, but also the organic components, such as DES and CH_3_CO_2_Li. The organic components, especially DES, play an important role on transferring Li-ion favorably, similar to the SEI from the viewpoint of structure and function. The related oxidation reactions of CH_3_CH_2_· radical are:

2 $$2\;{\mathrm{CH}}_3{\mathrm{CH}}_2\cdot + \, 1.5\;{\mathrm{O}}_2 \to 2\;{\mathrm{CH}}_3{\mathrm{CHO}} + {\mathrm{H}}_2{\mathrm{O}}$$

3 $${\mathrm{CH}}_3{\mathrm{CHO}} + {\mathrm{O}}_2^ - \to {\mathrm{CH}}_3{\mathrm{CO}}\cdot \; + \, {\mathrm{HO}}_2^ -$$

4 $$2\;{\mathrm{CH}}_3{\mathrm{CO}}\cdot \; + \, {\mathrm{O}}_2 + 2\;{\mathrm{Li}} \to 2\;{\mathrm{CH}}_3{\mathrm{CO}}_2{\mathrm{Li}}$$

For the neutral DES molecules, the GC-MS and XPS results have demonstrated that they still remain on the surface of Li anode in the TESI-containing Li−O_2_ cell after 60 cycles. However, partial DES molecules could be transformed into Li_2_S under the reduction of Li metal during repeated cycling. Generally, Li_2_S is mainly derived from the decomposition of the Li salt, as displayed in the LiI-containing cells. The related reduction reactions of DES with Li metal may be:

5 $${\mathrm{CH}}_3{\mathrm{CH}}_2{\mathrm{SCH}}_2{\mathrm{CH}}_3\left( {{\mathrm{DES}}} \right) + {\mathrm{Li}} \to {\mathrm{CH}}_3{\mathrm{CH}}_2\cdot \; + \, {\mathrm{LiSCH}}_2{\mathrm{CH}}_3$$

6 $${\mathrm{LiSCH}}_2{\mathrm{CH}}_3 + {\mathrm{Li}} \to {\mathrm{CH}}_3{\mathrm{CH}}_2 \cdot + \ {\mathrm{Li}}_2{\mathrm{S}}$$

**Fig. 9:**
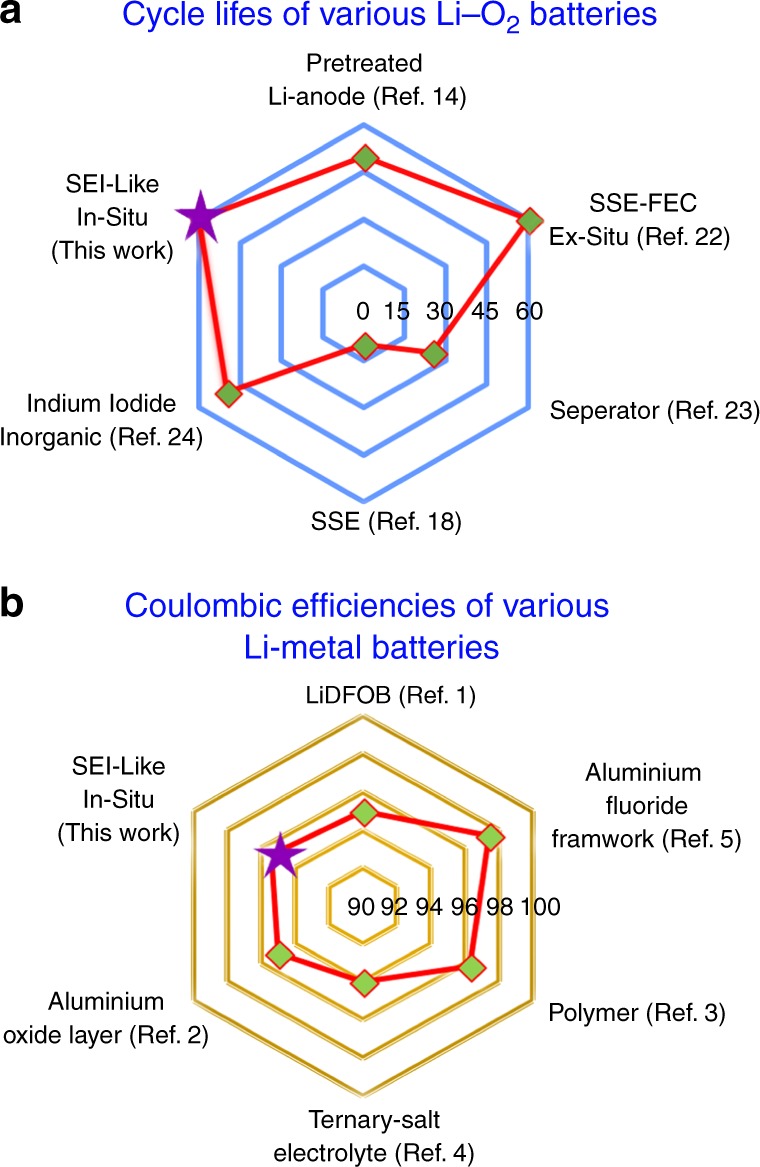
Summary of the performance of recently reported interfacial engineering methods improving the Li metal anode. **a** Cycling performance of various Li-O_2_ batteries. **b** Coulombic efficiency of various Li-metal batteries

It is relevant here to recall the issues of SEI component modification mentioned in the introduction section. Our results address these issues from the following aspects: (1) reductive ethyl detaching is a fundamental process to properly introduce organic components in an organic-inorganic hybrid SEI layer on Li anode; and (2) the oxidation process in the presence of O_2_ and its intermediates O_2_^−^ is essential to produce a SEI-like layer on the Li metal. In terms of battery performance, the TESI-containing Li–O_2_ cell exhibits improved cycling performance over the cells using other approaches to suppressing the shuttle effect of RMs, such as pre-treated lithium anode^14^, solid-state electrolytes^18,22^, modified separators^23^, and inorganic InI_3_^24^, as compared in Fig. 9a. Moreover, taking a broad view of interfacial engineering for next-generation lithium metal batteries, the CE of the lithium metal in this work is on par with those previously reported values (Fig. 9b)^1–5^. Giving the variety of organic iodide compounds, both ionic and covalent types, more research could be conducted to further improve lithium metal anode performance.

In summary, triethylsulfonium iodide is found to be chemically reduced on metallic Li anode, in situ forming a SEI-like layer between electrolyte and Li anode through reductive ethyl detaching and subsequent oxidation during the first few cycles of Li–O_2_ batteries. Different from the previously reported inorganic solid-state electrolytes or Li-In alloying protective layer, the SEI-like layer reported here is an organic and inorganic composite interface, which possesses high stability against the attack of soluble triiodide. More importantly, Li-ions can pass freely through the SEI-like layer such that deposition can take place underneath the layer without forming dendrites. Our results represent a new approach for RM developments based on bifunctional organic iodide. In particular, the reductive ethyl detaching can be used as a useful descriptor for screening candidate compounds for more bifunctional RMs which can produce SEI-like layer in the oxidation environment of Li–O_2_ batteries.

## Methods

### SWNT–SLG–IL gel

SWNTs (10 mg, Hipco Super pure) and SLG (1.36 mg, ACS Materials) were dispersed in 0.6 ml imidazolium ion-based ionic liquid (IL) of 1-ethyl-3-methylimidazolium ([C_2_C_1_im]) bis(tri-fluoromethylsulfonyl) ([NTf_2_]) by ultrasonic dispersion^39^. The suspension was ground together with 2 mg PTFE in an agate mortar for 10 min. With grinding, the SWNTs bundles and SLG sheets were bound by PTFE particles together into a spherical paste. After grinding continuously for about 30 min, the paste began to spread and become sticky. The subsequent grinding up to 60 min turned the paste to a viscous gel, accompanied by a visible volume expansion^39^.

### SWNT-SLG-IL gel-derived air cathodes

The as-prepared SWNT-SLG-IL gel was casted onto round-shape carbon paper with a diameter of 12 mm. The carbon paper with SWNT-SLG-IL gel was immersed first in NMP solvent for 30 min for 2 times, and then dried at 60 °C for 24 h. The obtained solid film was immersed in a solvent of ethanol-acetonitrile mixture for 12 h and then dried at 110 °C for 12 h. By the 2-step extraction method, IL was removed completely from the SWNT-SLG-IL gel. The finally obtained solid film was used as SWNT-SLG-IL gel-derived air cathode. The loading area of the SWNT-SLG gel-derived air cathodes was a square of 0.25 cm^−2^ on a carbon paper current collector. The loading weight of the SWNT-SLG was ~0.11 mg.

### Li anode

A 0.2-mm-thick Li foil was cut into a disc of 12.0 mm in diameter, and then pressed onto a stainless steel spacer in Li–O_2_ cells.

### Cu foil

A 0.25-mm-thick Cu foil was cut into a disc of 10.0 mm in diameter.

### Electrolyte and separator

A TEGDME-based electrolyte consists of Li salt of 1 M bis (trifluoromethane sulfonyl) imide (LiTFSI). 50 mM Triethylsulfonium Iodide (TESI) or lithium iodide (LiI) was added as RMs. The amount of the electrolyte was 60 µL, immersed in a Waterman GF/C glass fiber separator.

### Assembling Li–O_2_ cell

Two-electrode cells configuration using CR2032 coin-type cells with holes for O_2_ access was employed and assembled in an Ar-filled glove box with O_2_ and H_2_O content below 0.5 ppm. The Li foil and air cathode were the working electrodes. After assembling, the cells were operated in 1.0 mbar of pure O_2_.

### Assembling Li | Cu half-cell (in the presence of O_2_)

Two-electrode cells configuration using CR2032 coin-type cells with holes for O_2_ access was employed and assembled. The Li foil and Cu foil were the working electrodes. After assembling, the cells were operated in 1.0 mbar of pure O_2_.

### Assembling Li | Cu half-cell

Two-electrode cells configuration using standard CR2025 coin-type cells was employed and assembled. The Li foil and Cu foil were the working electrodes. After assembling, the cells were operated in air.

### Density functional theory calculation

Our calculations were based on the density functional theory (DFT) implemented in the VASP program^40^ using a planewave basis set. The kinetic cutoff energy for the planewaves was set to 30 Ry. The ionic cores were represented by the projector-augmented wave (PAW) potentials^41^. We used the Perdew-Burke-Ernzerhof (PBE) exchange-correlation functional^42^. Structural optimizations were carried out until the force on each atom was smaller than 0.5 mRy/Bohr.

### Electrochemical measurement

The current density was constant with a lower voltage limit of 2.0 V (vs. Li/Li^+^) and upper limit of 4.0 V (vs. Li/Li^+^) at 25 °C after a 3 h rest period. All the specific capacities were calculated by normalizing with the weight of the SWNTs-SLG. The electrochemical tests at 25 °C charge/discharge were performed using a Land battery tester (LAND CT2001) and Autolab instruments. The EIS measurement was performed in a frequency range from 10^6^ to 10^−1 ^Hz under amplitude of 10 mV using Autolab 84640 electrochemical workstation (Metrohm Autolab B.V., The Netherlands). The equivalent circuit used for fitting the EIS spectra is from a previous publication^43^.

### Sample preparation

All samples for analyses were prepared in the argon-filled glove box, washed by 1,2-dimethoxyethane (anhydrous, Sigma) for 0.5 h and then dried for 2 h. A special transfer system was employed to transfer the samples from the glove box to the analyses system without being exposed to air.

### XRD analysis

X-ray powder diffraction (Rigaku) was used to analyze the crystalline structure of the air cathode after cycling.

### SEM analyses

The Li anodes and cathodes after cycling were analyzed by the field emission scanning electron microscope (FESEM JSM-4800F) for micrograph observation.

### FTIR analysis

The Li anodes after cycling were analyzed by Fourier transform infrared (FTIR, JASCO FT/IR-6200). The samples were ground together with KBr and pressed into pellets.

### Raman analysis

Raman spectra were recorded with a Raman microspectrometer (Invia Renishaw, UK) using the 532 nm line of a semiconductor laser at room temperature.

### XPS analysis

The Li anodes after cycling were analyzed by the X-ray photoelectron spectroscopy (XPS, Thermo Fisher Scientific ESCALAB 250).

### GC-MS analysis

The analysis was performed on a Trace 1300 gas chromatograph coupled to a TSQ 8000 Evo triple quadrupole mass spectrometer and a TriPlus 300 autosampler (Thermo Fisher, Austin, TX, USA). The Li anode of the Li–O_2_ cell with 50 mM TESI after 60 cycles was prepared in the argon-filled glove box. The Li anode was put into a 20 ml glass sample container. The container was transferred from the glove box to the GC-MS analysis system and heated at 90 °C for 20 min to vaporize DES. 1000 μl of headspace was immediately sampled using a gas syringe and injected in the ion-trap GC-MS, equipped with capillary column (TG-5, 30 m × 0.25 mm × 0.25 μm) using helium as gas carrier. The temperature program for the GC run was: 25 °C initial column temperature, 10 °C min^−1^ to 120 °C, held for 5 minutes. High purity helium gas was used as the carrier gas and the pressure was kept constant at 75 kPa.

## Supplementary information

Supplementary Information

## Data Availability

The data that support the findings of this study are available from the corresponding author upon request.
