# Supplementary material for: Anode interfacial layer formation via reductive ethyl detaching of organic iodide in lithium–oxygen batteries
Source: Nat Commun. 2019 Aug 7;10:3543. doi: 10.1038/s41467-019-11544-8 (PMC6685988; doi:10.1038/s41467-019-11544-8)
Supplement: Supplementary file 1 — Supplementary Information [file 41467_2019_11544_MOESM1_ESM.pdf]

Supporting Information

Anode Interfacial Layer Formation via Reductive Ethyl Detaching of Organic Iodide in Lithium−Oxygen Batteries

Xiao-Ping Zhang et al.

Supplementary Figures

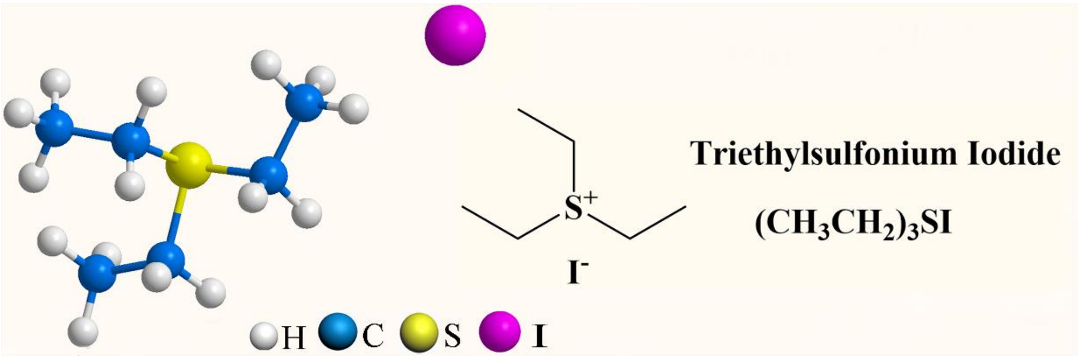

Supplementary Figure 1 | Triethylsulfonium Iodide molecular structural formula.

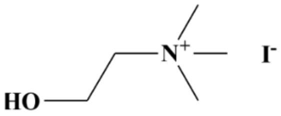

Choline Iodide

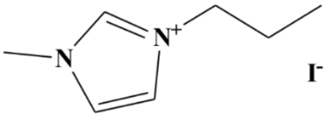

1-Methyl-3-Propylimidazolium Iodide

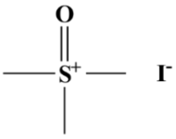

Tetramethylammonium Iodide

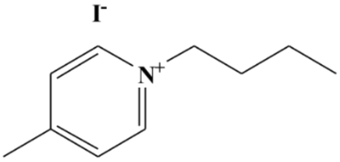

1-Butyl-4-methylpyridinium Iodide

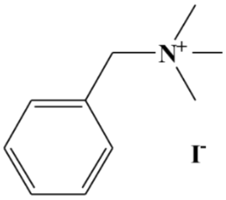

Benzyltrimethylammonium Iodide

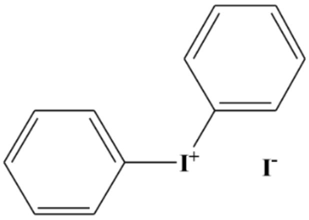

Diphenyliodonium Iodide

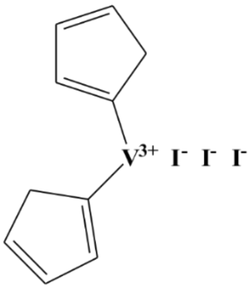

Bis(Cyclopentadienyl)Vanadium Iodide

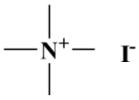

Tetramethylammonium Iodide

Supplementary Figure 2 | The partial list of organic iodides. The molecular formulas of organic iodides that possibly function as RMs.

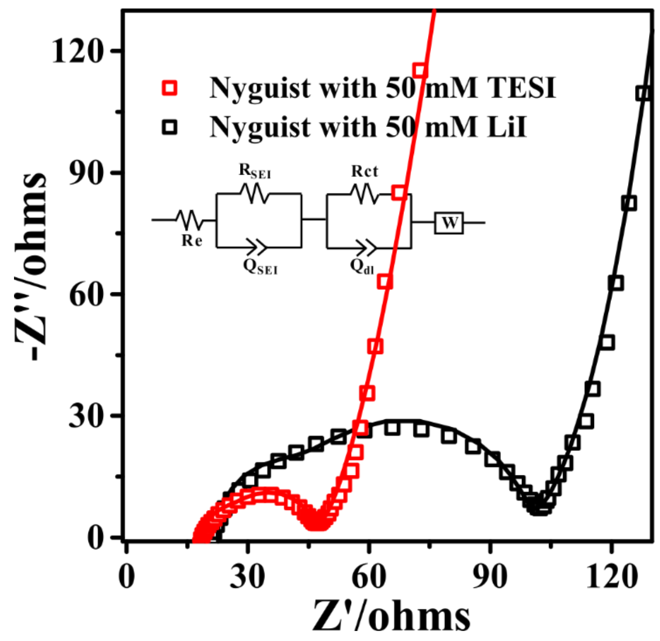

Supplementary Figure 3 | Electrochemical Impedance spectra of the Li−O_2 cell after 30 cycles with 50 mM TESI and 50 mM LiI. The Li−O_2 cells were reassembled with pristine SWNT–SLG air cathodes. EIS analysis using an equivalent circuit model (shown in the inset) and Nyquist plots of experimental data.

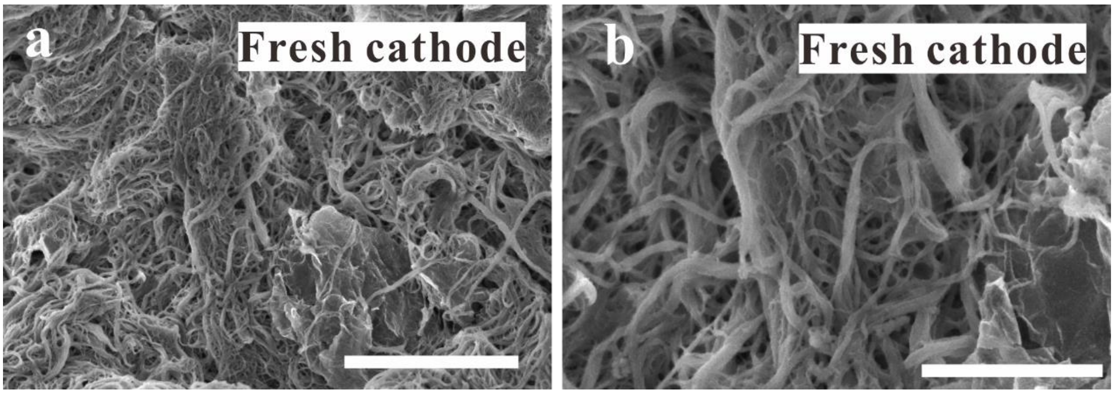

Supplementary Figure 4 | SEM images of fresh cathode. (a) The large-area view of the fresh cathode. Scale bar is 4 μm. (b) The small-area view of the fresh cathode. Scale bar is 1 μm.

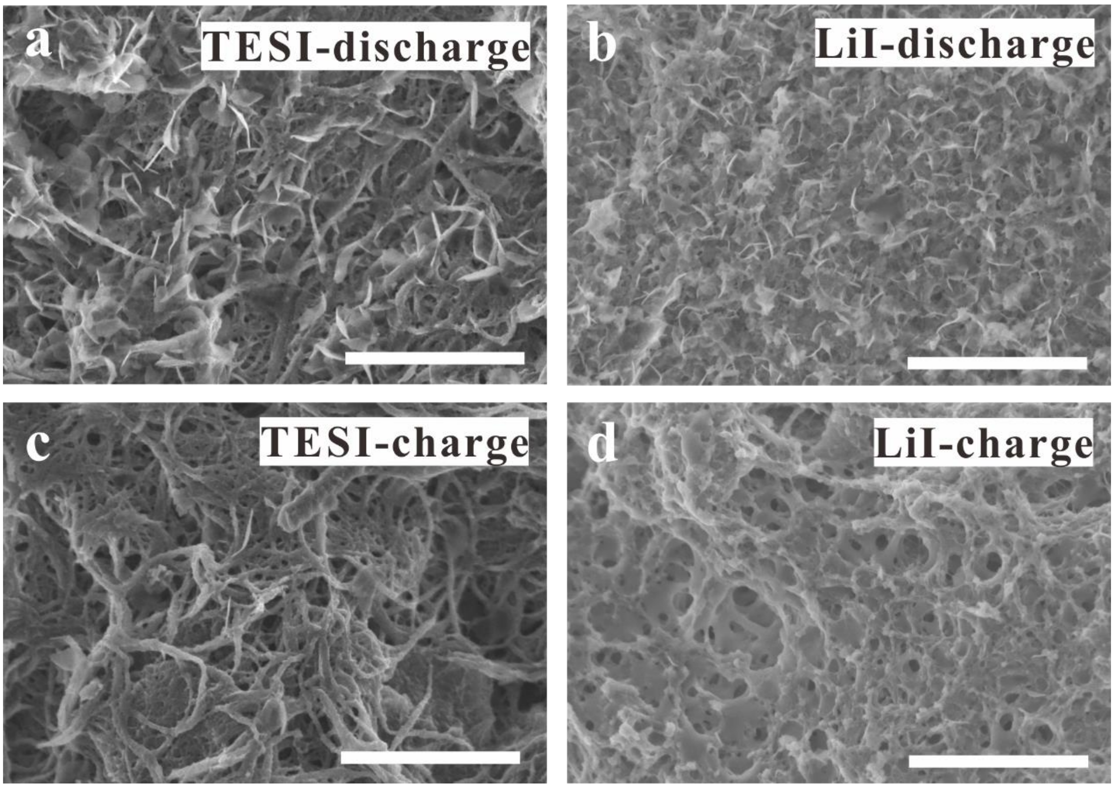

Supplementary Figure 5 | SEM images of the air cathodes after cycling in $Li–O_2$ cells. (**a**) SEM image of the air cathode discharged after 60 cycles with 50 mM TESI additive. Scale bar is 1 μm. **(b)** SEM image of the air cathode discharged after 30 cycles with 50 mM LiI additive. Scale bar is 1 μm. **(c)** SEM image of the air cathode charged after 60 cycles with 50 mM TESI additive. Scale bar is 1 μm. **(d)** SEM image of the air cathode charged after 30 cycles with 50 mM LiI additive. Scale bar is 1 μm.

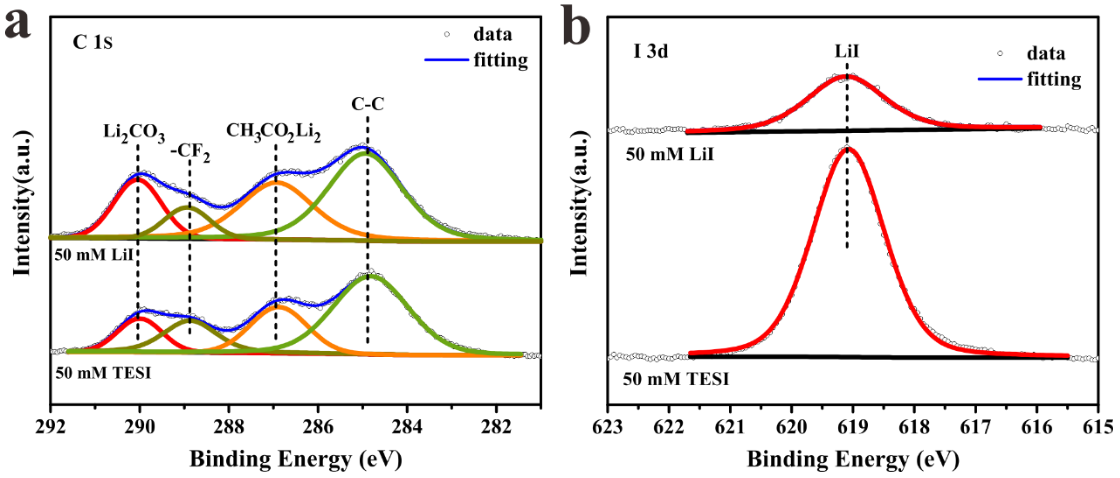

Supplementary Figure 6 | XPS spectra on the surface of the air cathodes from the Li–O_2 cells with 50 mM TESI after 60 cycles and 50 mM LiI additives after 30 cycles. (**a**) C 1s XPS spectra. (**b**) I 3d XPS spectra.

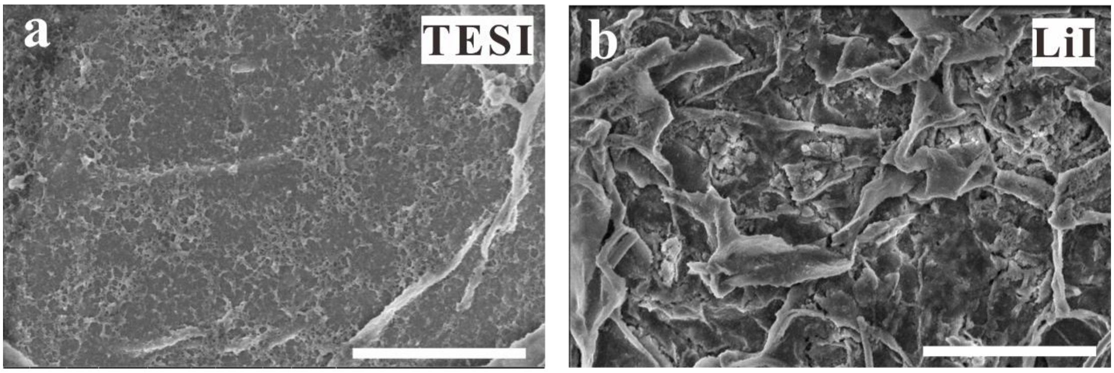

Supplementary Figure 7 | SEM images of the Li anodes after the first cycle in Li−O_2 cells. (a) With 50 mM TESI additive. Scale bar is 2 μm. (b) With 50 mM LiI additive. Scale bar is 2 μm.

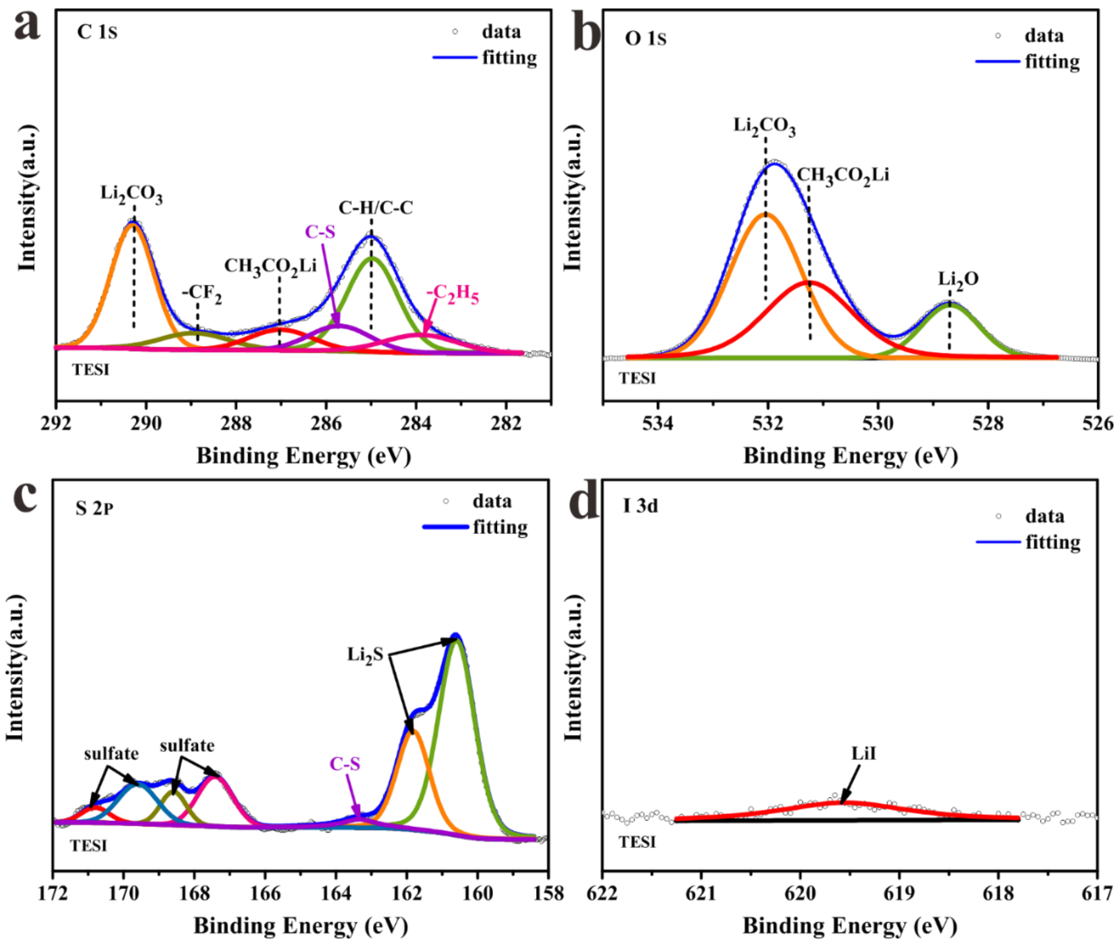

Supplementary Figure 8 | XPS spectra of the Li anode from the Li–O_2 cell with 50 mM TESI additive after 1 cycle. (a) C 1s XPS spectra. (b) O 1s XPS spectra. (c) S 2p XPS spectra. (d) I 3d XPS spectra.

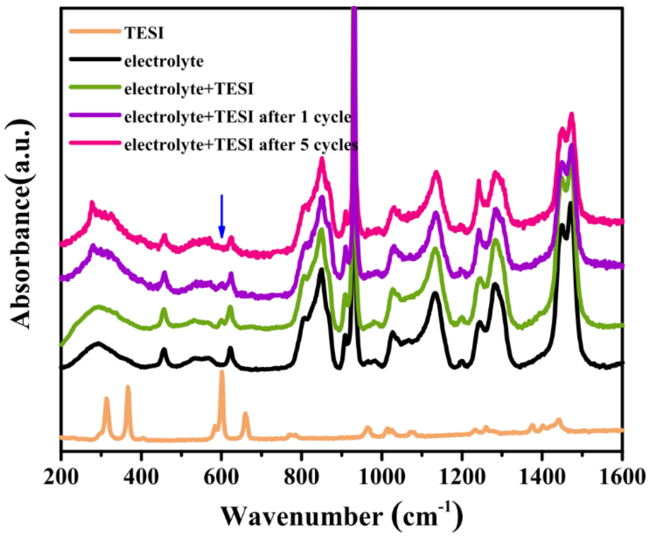

Supplementary Figure 9 | Raman spectra of TESI and electrolytes. Electrolyte is TEGDME-based, consisting of Li salt of 1 M Lithium bis (trifluoromethane sulfonyl) imide (LiTFSI). Spectrum from pure TESI is in orange, pure electrolyte is in black, electrolyte with 50mM TESI additive is in green, electrolyte with 50 mM TESI additive of the Li−O_2 cell after 1 cycle is in purple, and electrolyte with 50 mM TESI additive of the Li−O_2 cell after 5 cycles is in pink.

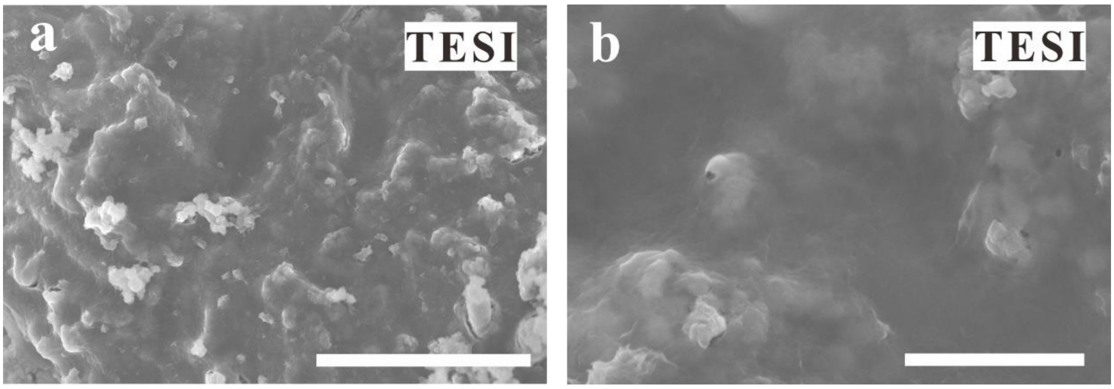

Supplementary Figure 10 | Surface analysis of the Li anode from Li | Cu cells in the presence of O_2 with 50 mM TESI additive. (**a**) The large-area view of the Li anode after 100 cycles. Scale bar is 5 μm. (**b**) The small-area view of the Li anode after 100 cycles. Scale bar is 1 μm.

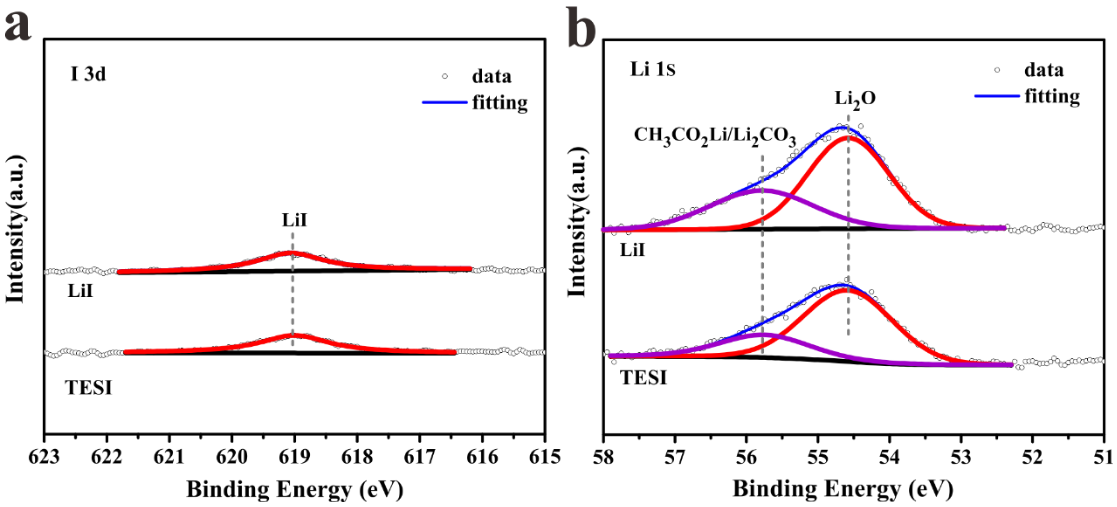

Supplementary Figure 11 | XPS spectra on the surface of the Li anodes from the Li | Cu cells in the presence of O_2 with 50 mM TESI and 50 mM LiI additives after 100 cycles. (**a**) I 3d XPS spectra of the Li anodes. (**b**) Li 1s XPS spectra of the Li anodes.

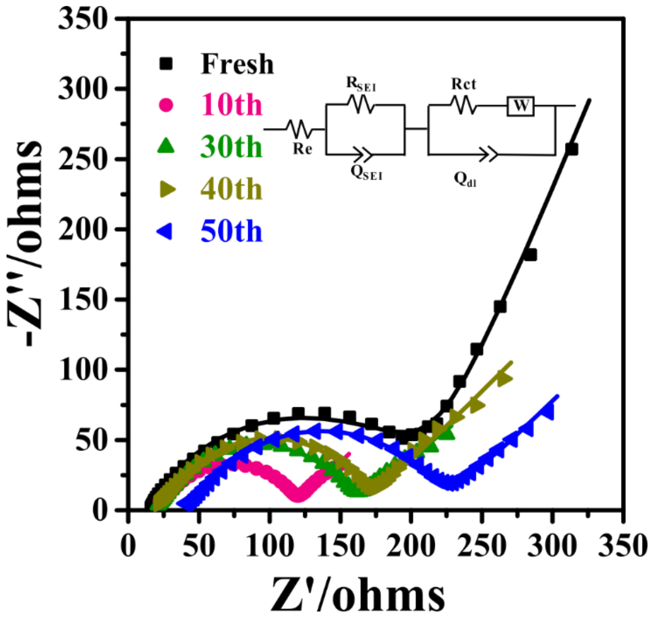

Supplementary Figure 12 | Electrochemical impedance spectra for 50 mM LiI-containing Li | Cu cell with different cycles. Inset figure shows the equivalent circuit.

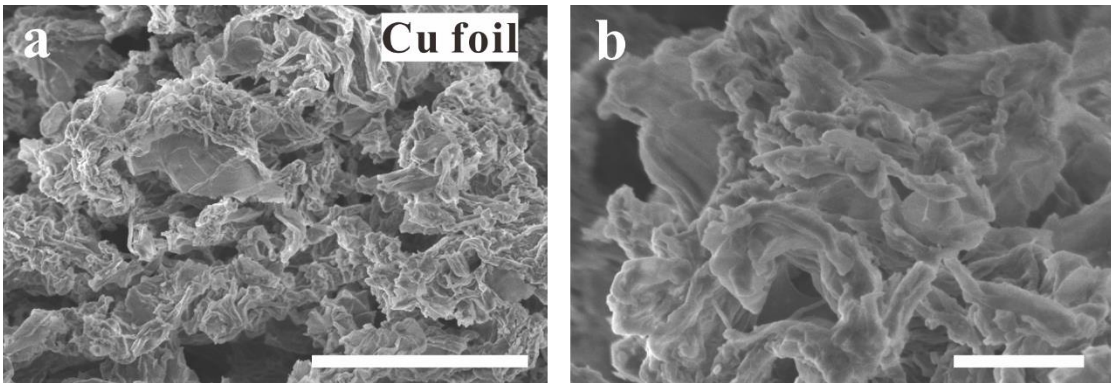

Supplementary Figure 13 | SEM images of the Cu foil from LiI-containing Li | Cu cell after 50 cycles. (a) The large-area of the Cu foil. Scale bar is 5 μm. (b) The small-area of the Cu foil. Scale bar is 1 μm.

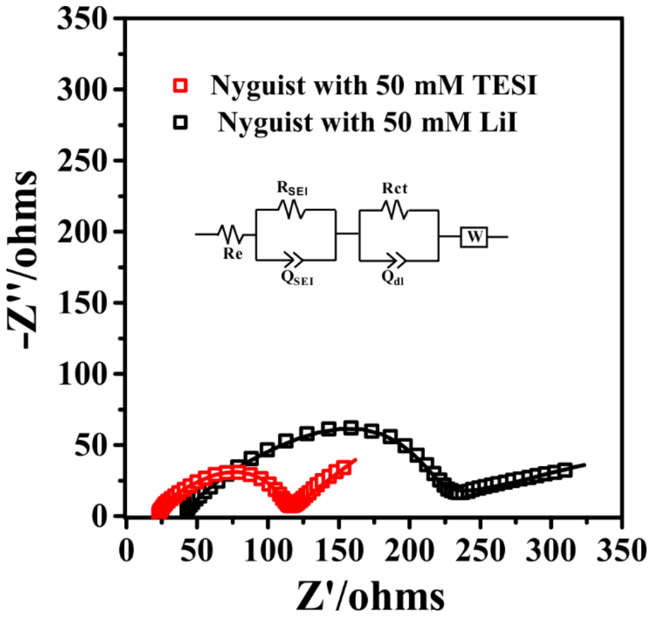

Supplementary Figure 14 | Impedance spectra of the Li | Cu cell after 80 cycles with 50 mM TESI or 50 mM LiI additive. EIS analysis using an equivalent circuit model (shown in the inset) and Nyquist plots of experimental data.

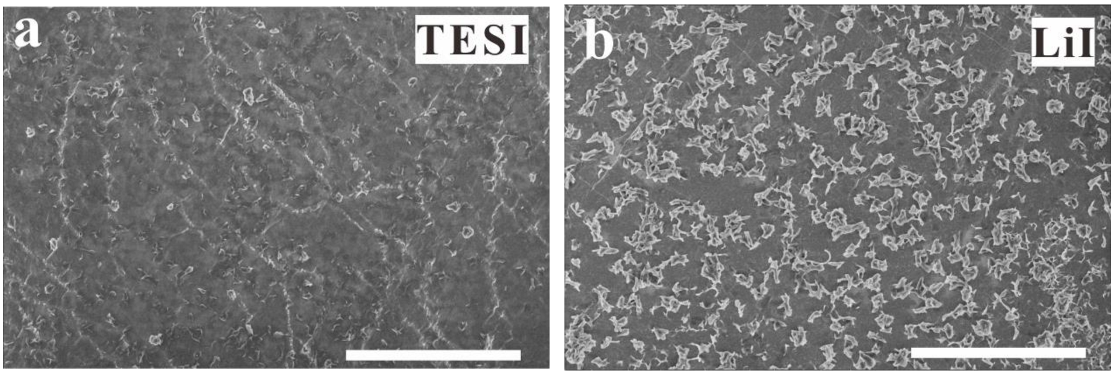

Supplementary Figure 15 | SEM images corresponding to *Fig. 8*. (a) With 50 mM TESI additive. Scale bar is 5 μm. (b) With 50 mM LiI additive. Scale bar is 5 μm.

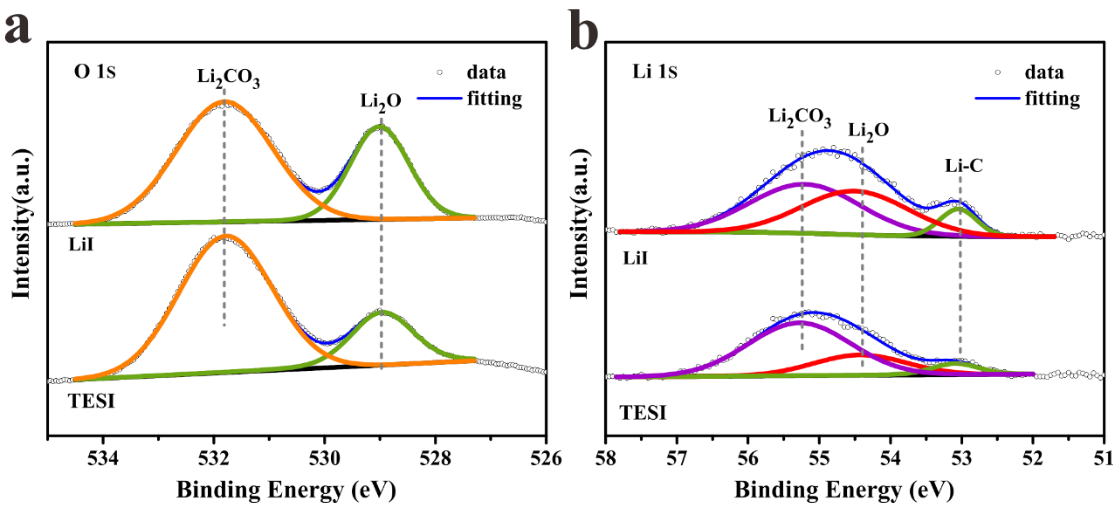

Supplementary Figure 16 | XPS spectra on the surface of the Li anodes from the Li | Cu cells with 50 mM TESI and 50 mM LiI additives after 80 cycles. (**a**) O 1s XPS spectra of the Li anodes. (**b**) Li 1s XPS spectra of the Li anodes.

Supplementary Tables

Supplementary Table 1 | EIS analysis corresponding to *Fig. 3c.* EIS analysis with Nyquist fitting parameters. R_e: equivalent series resistance for the electrolyte, current collectors and electrode materials. R_{SEI}: resistance derived from the interfacial contact and interface layers at the electrode surface. R_{ct}: charge transfer resistance at the interface of electrolyte and active materials.

	50 mM TESI	50 mM LiI
**R_e** (Ω cm^2)	13.49	37.28
**R_{SEI}** (Ω cm^2)	36.18	58.36
**R_{ct}** (Ω cm^2)	35.24	40.72

Supplementary Table 2 | EIS analysis corresponding to *Supplementary Figure 14*. EIS analysis with Nyquist fitting parameters.

	**50 mM TESI**	**50 mM LiI**
R_e (Ω cm^2)	21.94	28.62
R_{SEI} (Ω cm^2)	36.24	110.58
R_{ct} (Ω cm^2)	52.72	134.79

Supplementary Notes

Supplementary Note 1. Supplementary Figures 5a and 5b show typical flaky Li_2O_2 particles observed on the cathode during discharge in the TESI- and LiI-containing Li–O_2 cell. After charging, the decomposition of Li_2O_2 particles are complete, and the air cathode in the TESI-containing cell maintain pristine structure after 60 cycles (Supplementary Figure 5c). In contrast, the structure of the air cathode in the LiI-containing cell is destroyed after 30 cycles, which suggests that some of the carbon has decomposed (Supplementary Figure 5d).

Supplementary Note 2. XPS spectra on the surface of the air cathodes are shown in Supplementary Figure 6. Compared with the LiI-containing cell, the components on the surface of the cathode are the same as those in the TESI-containing cell.

Supplementary Note 3. XPS spectra on the surface of the Li anodes from the Li | Cu cells in the presence of O_2 are shown in Supplementary Figure 11. The amount of LiI is little on the surface of the Li anodes (Supplementary Figure 11a) This is because that LiI cannot be oxidized to I_3^-/I_2 at the low potential (< 3.55 V) in the Li | Cu cells and the shuttle effect of LiI is weaken. In Supplementary Figure 11b, the products on the surface of the Li anodes include CH_3CO_2Li, Li_2CO_3 and Li_2O, and these results are consistent with the O 1s and C1s results.

Supplementary Note 4. XPS spectra on the surface of the Li anodes from the Li | Cu cells are shown in Supplementary Figure 16. The products on the surface of the Li anodes include Li_2CO_3, Li_2O and Li–C bond, and the species related to C are consistent with the C 1s XPS results.
